# Knowledge gain and usage of knowledge learned during internet-based CBT treatment for adolescent depression - a qualitative study

**DOI:** 10.1186/s12888-020-02833-4

**Published:** 2020-09-10

**Authors:** Matilda Berg, Anna Malmquist, Alexander Rozental, Naira Topooco, Gerhard Andersson

**Affiliations:** 1 Department of Behavioural Sciences and Learning, Linköping University, SE-58183 Linköping, Sweden; 2 Department of Clinical Neuroscience, Karolinska Institute, Stockholm, Sweden; 3 UCL Great Ormond Street Institute of Child Health, London, UK; 4 Center for m2Health, Palo Alto, California, USA

**Keywords:** Memory of treatment, Adolescent depression, Qualitative methods

## Abstract

**Background:**

The role of explicit learning of treatment content in internet-based cognitive-behavioural treatment (ICBT) is an emerging field of research. The objective of this study was to explore clients experiences of their ICBT treatment for depression with a focus on knowledge gain and usage of knowledge learned during treatment.

**Methods:**

A strategic sample of ten adolescents, aged between 15 and 19 years, who had received ICBT for major depression within a clinical controlled trial were recruited for the study. Semi-structured interviews were conducted 6 months following trial completion. Data were transcribed and analysed using thematic analysis. The participants had a general adherence rate of 6–8 opened modules out of 8 possible.

**Results:**

Two main themes were identified; “Active agents of CBT” and “Passive agents of CBT”, with each theme consisting of three and two sub-themes. Active agents of CBT reflect a tendency to specifically remember and actively apply specific CBT principles in present life situations. Passive agents of CBT reflect a tendency to remember CBT treatment principles vaguely and express a passive or reactive usage of learned therapy content.

**Conclusion:**

The findings suggest that young clients can remember and apply CBT principles 6 months after their treatment. However, while experiencing benefits of treatment, clients recall and application of treatment strategies vary. The study emphasizes the importance of exploring client recall of CBT components and how valuable it is to explicitly remember contents of a treatment in order to improve and maintain improvement. Further studies on the role of knowledge and memory of ICBT for adolescent populations are warranted.

## Background

Internet-based Cognitive Behavioural Therapy (ICBT) is an increasingly established format to deliver Cognitive Behavioral Therapy (CBT), and has growing empirical support [1]. ICBT is usually delivered through an online platform, with the treatment content mainly being based on educational texts, and is commonly complemented with online therapist support [2, 3]. Based on the trials conducted so far, therapist-supported ICBT appears to be as effective as face-to-face CBT for a range of psychiatric and somatic disorders in adults, including depression [4].

The empirical support for ICBT in the treatment of depressed adolescents is also emerging. Depression is one of the main psychiatric problems among adolescents worldwide [5, 6], and ICBT can be an effective and feasible treatment for this population [7, 8]. Meta-analyses show promising effects of ICBT in terms of reducing symptoms of depression as well as anxiety and somatic symptoms in younger populations [9, 10]. However, little is known about the active components of ICBT and how treatment components affect adolescents in the long run. Adolescents experiencing a depressive episode unfortunately tend to relapse and this can lead to suicidal behaviour later in life as well as other adverse conditions such as substance misuse [5, 11]. It is thus important to evaluate whether and how ICBT can lead to sustainable effects that may prevent recurrence of depression and later problems in adulthood.

One potentially important outcome of ICBT could be what adolescent clients actually learn during therapy and later on remember and/or apply. Both might have implications for relapse prevention. However, little is known about how clients experience ICBT in terms of gained and applied knowledge [2, 12]. Some research has suggested that a focus on learning and memory of treatment content is needed in order to achieve successful treatment outcomes [12, 13]. It has also been argued that better memory of therapeutic content learned in treatment can help to prevent the occurrence of new subsequent depression episodes [12, 13]. Importantly, it is the explicit aspects of learning and memory that is referred to. Explicit forms of learning outcomes can be contrasted against implicit forms of learning outcomes, both of which can be important as outcomes of psychological treatments. Explicit learning and memory is information and experiences that we can actively and intentionally retrieve whereas implicit learning and memories can affect our behaviour and performance without our awareness, one example being motor skills learning [14]. By supporting clients’ explicit learning and memory of treatment content, the hope is that the principles and concepts covered in treatment will stay and thus help clients when they face new challenging life-situations later on in life [12, 13]. CBT (as well as ICBT) emphasize educational components and to engage clients in new adaptive learning experiences [15]. CBT includes concepts and skills that are regarded as important for the client to remember and continuously implement in everyday life, such as cognitive restructuring (identifying and challenging negative thoughts) and exposure (identifying and challenging fears). The importance of evaluating what clients learn and how they apply treatment content is underlined by research showing that that strategies that aim the enhance learning and memory can have a positive effect on treatment outcome but also that patients can forget the information and recommendations provided in mental health interventions leading to less improvement in outcomes [12, 13, 16].

Explicit learning outcomes of treatment can be particularly interesting to evaluate in ICBT since the treatment format can be viewed as a form of online education, mainly based on educational texts [3]. The aim is to provide clients with information that can help them make valuable and meaningful changes in cognitions and behaviours later on in their everyday life. ICBT has been described as an ideal context when studying learning and memory in psychological treatments [12]. As ICBT is highly structured and provide equal information to all participants (educational texts), it can be a suitable treatment format to study when evaluating what client actually gain from treatment. However, few studies have evaluated what clients actually learn, qualitatively speaking, and the processes behind remembering treatment content. Thus, we know little about whether ICBT achieves one of its indented goals, namely to educate patients with strategies and concepts that can help them in their everyday life, not only during but also after treatment. We also know little about how clients remember and use the rationales and principles explained in the treatment modules or about the personal insights connected to treatment content.

A few studies have investigated CBT outcomes with regards to learning and memory by using recall tasks, i.e., asking freely about what clients remember of their treatment [13], and how they apply treatment content, comparing the answers with a list of treatment components that are included in the treatment manual [13, 14]. In ICBT, some studies have used recognition tasks by presenting multiple choice tests when evaluating explicit knowledge gain during treatment [17–19]. This way of measuring learning and memory has some advantages but does not necessarily capture the range of gained knowledge or the unique impact of different treatment principles or how clients relate to them. Qualitative research methods can be helpful to gain a more detailed understanding of how content in ICBT can be remembered, and help identify the processes behind how knowledge is used and applied after treatment. There are studies that have explored patients experiences of ICBT using qualitative methods [20–22]. Such studies have however not yet focused on adolescents [11, 23], and to the best of our knowledge only one study (with adults) included a qualitative analysis of what they remembered from the treatment content as a part of analysis of ICBT impact [24]. Thus there is a need to investigate what adolescents with primary depression learn, remember and apply following a therapist-guided ICBT treatment.

In sum, little is known about what moderates or mediates treatment outcome in ICBT [9, 10] and few studies have evaluated the effects of ICBT with adolescent in a long-term perspective. This study used a qualitative method to explore adolescents experiences of ICBT, targeting explicit knowledge of CBT skills and the application of CBT knowledge. Participants were interviewed 6 months after the completion of ICBT as part of a depression trial.

## Method

The study was part of a controlled trial [8] that was approved by The Research Ethics Board in Linköping, Sweden (Reg. no. 2014/427–31). Participants were not offered any financial compensation for treatment or assessment completion.

### Participants

Participants were recruited from a randomized controlled trial on ICBT for adolescent depression that included 70 participants aged 15–19 years with mild to moderate depression [8]. They were recruited for the original trial via social media posts, and via information distributed to schools, youth centers, and clinics across Sweden (for further details about inclusion and exclusion criteria, please see Topooco et al. [8]). The present study used a strategic sampling based on interest, i.e., including those of participants who in connection with the post-treatment assessment over phone agreed to be contacted for a follow-up interview about their treatment experiences. Thus, participants provided informed consent at trial initiation and renewed consent was obtained by phone when explaining that the purpose of the interview was to ask about their learning experiences of therapy. Among participants who expressed interest to participate (*n* = 19), 11 gave verbal informed consent to participate in an audiotaped phone interview. Eight participants could either not be reached, or declined to participate for reasons such as lack of time or feeling uncomfortable to talk over telephone when contacted for the interview. Five of them were initially in the treatment group and three of them were in the control group. One participant was excluded from the analysis since she only had opened one module.

The ten participants included in the study had a mean age of 17.6 years (range 15–19). Nine were females and one was a male in line with the larger sample. Nine were initially in the original treatment group and one in the control group (controls had received treatment immediately following post-treatment assessment). Table 1 presents the characteristics of the ten study participants.

**Table 1:** Characteristics of participants

Participant	Age^a^	Gender	BDI^b^ pre treatment	BDI post treatment	Number of opened modules	Initial treatment condition^c^
Elise^d^	17–19	Female	47	15	8	Treatment
Emilie	17–19	Female	34	3	8	Treatment
Hanna	17–19	Female	27	20	6	Treatment
Amelia	17–19	Female	42	16	7	Treatment
Maria	15–17	Female	22	18	3	Treatment
Evelina	16–18	Female	18	15	7	Treatment
Julia	17–19	Female	38	23	7	Treatment
Clara	17–19	Female	29	26	6	Control
Alice	15–17	Female	27	3	6	Treatment
Jacob	16–18	Male	28	31	8	Treatment

^a^Age is presented in an approximate range to hide the exact age of the participants

^b^*BDI* Beck Depression Inventory. Scores range between 0 and 63. A total score between 14 and 19 points indicates mild depression, 20–28 points moderate depression, and 29–63 severe depression

^c^All participants in the control group received treatment after treating the initial treatment group

^d^The names in the Table are pseudonyms to secure the anonymity of the participants

### Internet-based cognitive behaviour therapy (ICBT)

The internet-administrated treatment program included 8 ICBT modules, and 8 individual therapist sessions delivered via chat; the entire intervention lasted 8 weeks and was available provided through a secure, participant-specific homepage requiring two-step authorization. Modules included established CBT-principles of behavioural activation, cognitive restructuring, affect regulation, and exposure, and associated home-work assignments. Clients had regular contact with a therapist who provided feedback and support on treatment-related exercises. Further, weekly 45-min long chat-sessions with therapist were included as part of the treatment protocol. The chat-sessions consisted of real-time therapist support via text-messages and focused on content and exercise completion within the treatment modules. The control group was an attention control condition that included access to the platform and an assigned therapist. In this way, the participants in the control group could be monitored and contacted for non-specific counselling if their symptoms levels deteriorated during the waiting period. As stated the attention control group received treatment immediately after the treatment group. Overall, the treatment had a large reducing effect on depressive outcomes (intention-to-treat analysis: *d* = 0.86, 95% CI 0.37–1.35). A more in-depth description about the ICBT treatment and the results is presented elsewhere [8].

### Procedure and qualitative method

The participants were interviewed over telephone within 6 months after the treatment trial had ended. One female psychologist (the first author) conducted the interviews. The interviews ranged between 12 to 29 min in length, with a mean length of 18 min. The interviewer had no previous contact with the participants. All interviews were recorded and subsequently transcribed. Participants were informed about the study, the procedure, and the aim to further understand the effects and experiences of treatment in a long-term perspective. They were informed that their information would be presented in an anonymous format, not allowing identification. As a consequence we use pseudonyms to secure the anonymity of the participants.

The interview guide was designed by the research group and included four broad open-ended questions asking what the clients had learned from treatment and how they applied and experienced the use of potentially acquired knowledge. See the interview guide in Appendix A. We analysed what the clients could tell about their learning experiences, interpreted to what extent they experienced learning and usage of treatment knowledge, and also how much they could remember of specific CBT principles. If the participants responded vaguely to the questions, they were asked follow-up questions, such as “*Give yourself a moment. If you think back, you went through a treatment containing modules with texts and exercises each week. Did you learn something from these modules*?”, or “*Could you give me a specific example?”* in order to facilitate recall. The number of follow-up questions needed to remember treatment content was taken into consideration when analysing the material, e.g., to what extent the participants needed further questions in order to recall treatment principles and how easily and concretely they could access the acquired CBT knowledge. Thus, we considered whether answers came after open-ended questions only and to what extent participants needed follow-up questions.

The transcriptions were analyzed using thematic analysis. Thematic analysis is a method for identifying and analyzing meaningful patterns (themes) across a written data set, in relation to the research question(s) [25]. The analysis generally proceeds in structured phases. First, the researcher(s) become familiar with the data and generate initial codes or keywords. The codes capture statements (units) within the transcripts that seem to reflect a meaningful concept. Then, the researcher(s) search for emerging themes among the different units. This is an iterative process where all the themes are discussed and re-reviewed, assuring that they still fit the data and relates to the research question in a meaningful way. The final steps are to define, label, and report the themes. Here, in order to further refine the results, each theme was differentiated into sub-themes. Importantly, this study used a theoretical thematic analytic approach [25]. The thematic analysis was theoretical since it was guided by theoretical concepts from CBT, searching for pre-determined information about CBT-principles and facts within the material.

All interviews were conducted, transcribed and initially coded by one researcher (the first author). Further analyses, as in searching and building of meaningful themes and sub-themes, were made together with two fellow researchers (the second and last author). The first author (M.B) had training in qualitative methods and three of the authors had previously worked with qualitative methodology and published qualitative studies (A.M, A. R, G.A).

## Results

### Overall results

The results showed that all participants could to some degree remember, relate to, and use their knowledge about CBT-principles, when interviewed 6 months after the trial had ended. Two main overarching themes were identified regarding how the participants described knowledge gain and usage of treatment content. First, several adolescents (*n* = 6) described how they explicitly remembered and actively related to CBT-principles in their everyday lives (e.g. “*[that’s] the one I always use, on a daily basis, you know*”), here defined as a theme of “active agents of CBT”. These participants could easily give specific examples of concrete CBT strategies and its utilization, such as exposure, worry-time, behavioural activation or sleep restriction. In general, they needed few or no follow-up questions in order to remember CBT principles, thus the treatment content seamed easily available to them. They also expressed an active use of the strategies learned in their current lives and applied the treatment material. The other overarching theme described how participants viewed themselves more as passive or reactive agents of the principles learned in therapy, here defined as a theme of “passive agency in CBT” (e.g. “*It shows up sometimes, I guess, like, when you really need it*”). These participants (*n* = 4) needed more follow-up questions and help from the interviewer in order to remember treatment content, and gave more vague examples of gained and applied knowledge. These participants described an experience of using CBT-strategies, but more in terms of being a possible support when feeling worse. Treatment outcome was described as implicit changes in knowledge over time, rather than present use of explicit CBT-tasks.

Below follows a more detailed description of the results. The excerpts have been selected since they were found to be representative of the data as a whole, illustrating the essence in the identified themes. An overview of the results are presented in Table 2.

**Table:** Table 2

Themes	Subthemes	Examples of quotes	Codes
**Active agents of CBT principles**	Concrete CBT-strategies	*“I guess the most important thing is thinking about the consequences. Like thinking about the long-term consequences of skipping school for example, now I can understand why it’s not so nice doing certain things even if it is a relief there and then when you do it. I had not realised that.. like.. before.. that.. yeah.. that it may not be so good for you in the long run”.* Participant [Evelina^a^]	Exposure Behavioural activation Long-term consequences Sleep restriction Affect-regulation Worrying-time Avoidance strategies
	Active approach during and after treatment	*“Well I cannot say that the anxiety for doing things has, like disappeared, the things I used to get anxious about still makes me anxious, like I have to work with it actively. Not giving up. Keep going.”* Participant [Clara]	Applying it in real life Continuous work Deliberate practice Active reflection Taking time Doing home-work Don’t give up
	Experience of insight	*“Those texts said so much about yourself, things I did not know about, you know, like they gave me new perspectives on how I felt and why I felt the way I felt, I knew I was feeling bad, but not why, so I just kept going, and then I learned a lot about why, that there was a reason behind it, like it was not my fault and that this is how things can be, and like this is what you can do to improve it.”* Participant [Alice]	Aha-moments Finding an explanation Understanding triggers Thoughts and emotions are manageable Eye-opener Faith in treatment
**Passive agents of CBT principles**	Vague general strategies	*“I learned about attention.. like.. sure you can like work with your issues, but you shouldn’t put too much energy into things that you cannot change anyway.”* Participant [Jacob]	Vague CBT-principles Learning about what works for oneself Changed attitude to oneself Less controlled by symptoms
	Passive or reactive approach	*“It’s not something I really use, but more like, if you start feeling worse again you can go back and check the material, like maybe trying something out again.”* Participant [Amelia]	Intuitive knowledge Implicit knowledge Using CBT when feeling worse Life continues Shrugging

^a^The names in the Table are pseudonyms to secure the anonymity of the participants

### Theme. Active agents of CBT principles

Three different sub-themes were derived from the interviews within the theme of participants as active agents of CBT-principles. The first sub-theme captured how these participants recalled CBT-principles explicitly during the interview. The second sub-theme captured an active application of gained knowledge about CBT, both during treatment and in their current lives. Finally, the third sub-theme highlighted a shared experience of insights achieved from the therapy and thus how knowledge gained from the modules changed the participants way of relating to themselves and their symptoms.

#### Sub-theme: explicit recall of CBT principles

Several participants spontaneously gave concrete examples of CBT principles learned during treatment. When asked, they generated explicit answers about acquired knowledge, as if it was easily accessible from their memory and usually thought upon. Even if some participants also expressed how knowledge from the treatment had become intuitive, and was not always present, they could recall at least one concrete example of a CBT exercise, concept, or principle that they actively used and applied 6 months after treatment completion. One of the participants, a girl about 17 years old, described her continuous use of exposure, i.e.*,* the principle of approaching and challenging your fears in CBT:

“***Stay in*** *situations that feel scary, you know, like when I am in a crowded place, that’s tough for me, and I kind of want to get out of there, I try to like stay in that place anyway, and kind of evaluate it.. like will this be really scary or not? ..[ … ].. and like if I can choose between, like phoning or sending a text, I’ll call, you know, expose myself to it..[ … ].. and if I get anxious about something, well, that feeling will pass, you know, it won’t keep growing, if you stay in that place, then the feeling goes away, it fades..”*

Participant (Elise)

This participant described the core principles of exposure easily, without any need of follow-up questions from the interviewer. She described concretely and with enthusiasm how this CBT strategy had helped her to manage new difficult situations, and how relevant this was for her everyday functioning. Another participant, a girl about 18 years old, similarly described her continuous and daily use of the module focusing on sleeping problems:

“..like not to be in your bed except when you are actually going to sleep, things like that, that makes it easier to fall asleep. That’s when the exercises and the texts about sleep definitely helped, without them it wouldn’t have worked for me. Yeah, when I think about it, really think about it, then it becomes easier to sleep, … [ … ]. Sleep [strategies from the sleep module] that’s the ones I always use, on a daily basis, you know”.

Participant (Hanna)

The quote shows how the participant explicitly recalled and concretely reflected on what she had learned from treatment. CBT-strategies for sleep were described as a commonly used and valuable skills in her everyday life. Importantly, when participants gave examples of explicit CBT-strategies, they were able to describe them correctly, i.e.*,* they did not give examples of incorrectly understood rationales or misunderstandings of psychoeducation. Further, the participants generally attributed the knowledge gained about these principles as important reasons for their improved well-being. Learning and using CBT principles had positively affected their ways of coping and made symptoms and difficulties more manageable.

#### Sub-theme: active approach during and after treatment

The second sub-theme highlights the experienced importance of actively processing and applying what participants had learned from the CBT-material. This was evident in how the they described how they had approached the treatment modules while going through treatment. One of the clients, a girl about 18 years old, described how she had realized the importance of not only reading and understanding the material, but also to actively use it in order to achieve results:

“It became clear what you need to do, to realise that you actually need to apply those things in real life for it to work. Yeah, I have to use the things that were written, somehow. I did not just read about it.”

Participant (Hanna)

The quote illustrates how the participant emphasized the importance of using the information and applying what she learned in order to benefit. A similar emphasis on active usage of the treatment was also reflected in descriptions of how modules were returned to and used again after the treatment period had ended. This can be illustrated by a quote from a female participant about 17 years old:

“Earlier, I always wound myself up, like, as soon as something gets just a bit stressful, I kind of panicked, but now I feel like I have some sort of tool to, like.. to manage the situation, I know that I can look back at the study and all of the things in it, which I’ve saved, and it feels like a comfort, in a way”

Participant (Alice)

The girl in the quote above expressed her continuous use of the treatment material in her present life. For her, the modules continued to be a helpful tool, and gave her a sense of security. Likewise, the theme captures a tendency to attribute therapeutic change to an active usage of treatment content during and after treatment.

#### Sub-theme: experiences of insight

The third sub-theme relates to insights derived from the specific treatment content, most often from the text in the modules. The sub-theme also involved the knowledge which had provided them with an increased capacity of labeling their thoughts and feelings as well as understanding triggers of distress. One girl about 17 years old described how the treatment material gave her new perspectives on her problems:

“Then I sort of got it, like wow, this is really great, that I really needed help with this, you tend to go a bit blind yourself.. and.. ah, there were quite a lot of those “aha- moments,” and things that surprised you, you know.”

Participant (Alice)

As illustrated in the quote, the treatment modules had resulted in some insights for the participants, had helped them clarify their problems and gain tools to deal with them in new ways. Reading the treatment material also seemed to normalize her thoughts and feelings. This is reflected in the quote below, expressed by a female participant (about 17 years old):

“So you read those modules and like oh god this is exactly the way I feel, here and here and here, and that you could actually treat it. It made sense, so weird, like there’s nothing wrong with me, like, so this is how things can be, it was so nice!”

Participant (Elise)

This participant shared her strong feeling of recognition while reading the texts and learning about CBT principles, and the quote above illustrates the relieving experienced. Knowledge about CBT helped her to understand her situation in a more constructive way, and brought a sense of hope and joy.

### Theme. Passive agents of CBT-principles

While some participants highlighted the need of an active approach and explicit use of CBT-strategies, a number of participants talked more about their learning process in a more passive manner. Two subthemes were differentiated from the overarching theme of passive agency. The first sub-theme captures a description of vague and general treatment strategies learned from treatment rather than specific CBT principles. The second sub-theme describe a passive approach when talking about the use of gained knowledge both during and after treatment completion.

#### Sub-theme: vague general treatment strategies

During the interviews, some participants responded vaguely when asked what they learned and applied from treatment. When asked more specific questions, some could describe CBT-principles but follow-up questions and support from the interviewer were needed in order to recall treatment content. Thus, specific CBT principles seemed less accessible and not as concretely utilized in their everyday life, in contrast to the theme of active agents of CBT-principles. One female participant (about 19 years old) exemplified this in the quote below:

“Well, like when you do not want to.. or if you want to.. like say something, I do it.. like I do in school or something..”

Participant (Amelia)

This quote illustrates the tendency to give vague responses to questions about learned and applied knowledge. The CBT-principle of trying to approach what you usually avoid is somewhat indirectly and implicitly noted in the response. However, it is clearly not as easily recalled or specifically described as in the responses described within the theme of active agents of CBT. Overall, the participants described their learning process in more generic terms. This can be illustrated by a quote from another female participant:

“Like, it feels like as if I got to know myself better. What works for me and what doesn’t, I can see what I need when I feel in a particular way, like when things happens I just kind of know what to do, like the body knows more than I do.. kind of.. I mean it is not so conscious, or back then it was, but now it’s more like.. yeah.. like ‘I just know’ without thinking.”

Participant (Emilie)

This quote illustrates how the treatment taught her new general perspectives and strategies to handle her life rather than explicitly mention any specific CBT principles. She expressed a feeling of an enhanced implicit knowledge about what works for her in different situations. Importantly, the theme reflects experiences of being helped by the treatment, but not in terms of explicitly recalling and expressing active use of CBT strategies, as in the theme of active agency.

#### Sub-theme: passive or reactive application of CBT principles

Further, the second theme showed how participants identified that they primarily used returned to CBT principles if they began to deteriorate in their symptoms. This reflects a more reactive and passive approach, in contrast to the expressed importance of continuously using and relating to treatment content within the theme of active application of CBT principles during and after treatment. One girl (about 19 years old) described this as below when asked about a specific example about how she uses knowledge from the treatment content today:

“Like, no I, well I guess it has happened a few times, but yeah it is more that I know that I have them [the modules] somewhere and if I need to think differently or something, I know I could just read them to remind myself, if.. yeah, you know, if I should start feeling worse about myself again.”

Participant (Maria)

The quote illustrates a tendency to use and apply treatment knowledge in a more reactive manner. Participants who contributed to this theme generally described how their everyday life just goes on, relying on that knowledge learned from treatment will be used or show up in times of need. This can be illustrated by a quote from a male participant (about 17 years old), when telling about how he applies learning experiences in his everyday life:

“.. well it’s not so clear for me, but more a change of personality, like, I think less about problems and more that things will be alright, that it’s not the end of the world, a bit more of that attitude than before.. more.. like.. ah.. it [CBT-principles] shows up sometimes, like when you really need it”

Participant (Jacob)

The participant shared a more passive approach to the utilization of treatment content, relying on the belief that knowledge gained from treatment will show up when required. Overall, the quotes in this theme captured a tendency to describe treatment success in terms of implicit changes in knowledge of over time, rather than actively relating to learned CBT-principles.

In sum, the clients experienced that they had learned important things during their treatment. However, the degree of specific knowledge about CBT principles and applied CBT-strategies varied. Some explicitly recalled concrete CBT principles and that they had gained insights they actively used in their everyday lives. Others related more vaguely and did not explicitly recall or exemplify the treatment content with CBT principles but rather described what they had learned in more general terms.

## Discussion

The overall aim of this study was to investigate adolescents’ experience of guided ICBT, with a focus on gained and applied knowledge 6 months after trial completion. Participants completed a semi-structured qualitative interview over phone.

The thematic analysis showed that all participants had some knowledge about CBT, but also revealed differences in how treatment content was remembered and applied. Two overarching themes and five sub-themes were identified. The two overarching themes were labelled *active agents* and *passive agents* of CBT principles. Conceptually, the main overarching themes can be understood as two ways of remembering and using treatment content. The theme of active agents of CBT pinpoints how some clients tend to remember more specific concepts and gained insights related to CBT, applying the knowledge actively during and continuously after treatment. They could recall one or several explicit examples of CBT principles and specify how they used the gained knowledge in their everyday life. One way to express this is that they *steer their own CBT boat,* managing the waves and currents with the tools. The theme of passive agents, on the other hand, showed how clients could relate to the treatment content but remember it in a more abstract way, passively or reactively relying on content of therapy to be used when they need them rather than actively applying them. A way to express this is like they use CBT principles as a *lifebuoy* when, or if, the sea becomes turbulent, trusting it to save them in times of need.

Importantly, the interviews were conducted with open-ended questions and can thus be viewed as a measure of free-recall. Answers on free-recall measures usually involve information that is accessed beyond directly asked questions [14]. Thus, responses to such questions do not only rely on familiarity of learned information as in recognition tasks but also require an active retrieval of learned material [14, 26]. We do not know, however, if the participants would have remembered more or differently if we had provided cues or questions specifically mentioning CBT principles. Further, we did not have access to participants actual behavior and do not know if other, fewer or more principles are applied in real-life situations.

The finding that clients accurately could recall content-specific techniques and modules after the treatment completion was also found in Halmetoja et al. [24] who partly studied what participants remembered from the treatment modules 4 years after ICBT in a qualitative study on adults with Social Anxiety Disorder. Halmentoja et al. [24] did not ask, however, about utilization of knowledge or analyzed differences in how the participants remembered treatment content. Thus, this study adds to the literature by showing that some clients can remember one or several concrete CBT strategies from treatment that they actively use, in line with the educational purpose of CBT. The analysis also shows that not everything is remembered of ICBT. Indeed, some clients rather seems to remember one strategy, that they experience as having a high impact in their everyday life. This is important to note when evaluating what clients remember in relation to treatment outcome. It might not be the total score or amount of remembered principles and strategies from treatment that is of relevance, but rather their unique impact or relevance for the unique person [13].

In contrast to the present study findings, Halmetoja et al. [24] did not report specific differences in remembered treatment content among participants or if they used their gained knowledge. The difference between how treatment content can be used and applied has been observed in other qualitative studies. Among adults who participated in ICBT for depression, Bendelin et al. [27] found a difference between clients who actively applied the material and clients who only read the treatment material. Bendelin et al. [27] related these different approaches to treatment outcome and found that the active appliers benefitted more from treatment than those who mainly just read the material. Enhanced benefits associated with active usage of treatment content was partly confirmed in this study since the active agents of CBT provided more specific knowledge derived from treatment and highlighted treatment content as the main reason for their treatment success.

Both overarching themes found in the present study (Active agents and passive agents) could hypothetically be viewed as two expressions of a successful treatment outcome. The theme of passive agents did reveal less concrete knowledge and insights related to CBT compared to active agents, but did still attribute their improved mental health to the treatment. This could possibly reflect a shift from explicit recall of treatment content to implicit or automatic knowledge use, i.e., that the skills learned in therapy had been automatized and affected performance and behaviour outside of conscious awareness [13, 14, 26]. The treatment material might be experienced as a natural part of their everyday life rather than remembered as CBT-principles learned from the treatment.

Nevertheless, the distinction pinpoints an interesting question for further investigation, namely that it is unclear to what extent recall of explicit knowledge of and insights related to CBT principles are needed for therapeutic change in ICBT. For some patients, the rationale and education about CBT principles might motivate them to participate and go through a treatment but does not necessarily need to be learned and remembered. Or, the lack of explicit learning of CBT-principles could be an issue to address further and highlights the need to actively support explicit memory of treatment content, as suggested by Harvey et al. [12]

The results presented here were explorative and preliminary and need to be validated in larger, quantitative trials. How clients remember and apply knowledge could provide further understanding of how to deliver and boost treatment content. It also calls attention to the unique way young people can engage in therapy and that some adolescents may experience benefits from therapy without explicitly remembering or using its content.

### Limitations

First, we only studied explicit knowledge gain and usage by using four open-ended questions. The questions used could be biased. It would be interesting to use specific cues woven into the questions and see how that affect the answers. Future studies would benefit from asking more specific questions about each module and the theme of the module, for instance behavioural activation and exposure. Alternatively, the interviewer could specifically ask the participants to list strategies learned in treatment to help them remember the content more clearly. It could also be interesting to provide respondents with different hypothetical scenarios and ask them to generate advices on how to manage the scenarios based on their knowledge about CBT. If asked to apply what they learned in relation to specific events participants might remember differently than we found in this study.

Second, like all qualitative studies, we need to be cautious about the degree to which our findings can be transferred to other settings [28], or transferred to the experience of young people other than those interviewed. The sample cannot be regarded as representative for adolescents who receive ICBT for depression. In addition, there is a risk that the participants involved in the interviews had a positive experience of ICBT or had a better mental health at the time of the interviews and thus gave a more positive picture of the treatment than other participants in the treatment would have done. If a patient feels better it might increase his or her motivation to participate in a post treatment interview.

While the possibility to generalize the results of qualitative study is limited [29, 30], the qualitative method used in this study was helpful in order to get a picture about how adolescents can remember treatment content. Hopefully, this can help us understand how young people can engage and use CBT-principles, and thus generate new ideas on how ICBT works and how it can be further studied in order to be optimally tailored for different patients.

As a third limitation, it could also be the case that the adolescents were polite and did not want to criticize us. Further, there was a variation in the adolescents’ habit or comfort in expressing themselves during the interviews, with some participants being quieter than others. It was a challenge to give all participants equal space and attention when conducting and reporting the results. Also, some patients might remember more than they are able to express due to nervousness. Differences in passive and active recall could possibly reflect differences in how confident or comfortable the respondents were in sharing their experiences, or differences in interview time. However, there was no obvious difference in word flow or confidence during the interview connected to the two overarching themes or length of the interview.

Importantly, one of the respondents was initially in the control group and thus participated in the interview about 4 months after treatment completion compared to respondents in the initial treatment group who participated 6 month after their treatment period. If participants had been interviewed closer to treatment completion, strategies learned during treatment might have been easier to remember. However, some participants from the initial treatment group did remember equally or more about CBT than the participant from the original control group.

Further, the BDI was not administered in association with the interviews and we therefore do not know how the participants rated their symptom levels at the time of the interview. Also, only one male participated, which reflects the preponderance of females in research on ICBT for depression and adolescents [7, 8], and shows the need to recruit more males in future studies.

Another limitation involves the potential interviewer bias as the researcher that interviewed participants (M.B) had worked with the treatment material. However, a person with no experience of ICBT was also involved in the analysis (A.M) and the two authors continuously reflected over the process and how prior knowledge could affect the analysis. In addition, in order to find and match the interview material in an ICBT-framework, one could argue that the researcher conducting the analysis need to have at least basic knowledge about the treatment content. By knowing about the ICBT program, the researcher knows what the clients could remember in relation to the treatment content and to what extent their answers reflect CBT strategies.

Finally, the quotes could possibly be interpreted in other ways than here since the focus was on if and how the adolescents could recall CBT principles. As mentioned, this is a preliminary study and the results should be interpreted with caution. The research should be complemented with quantitaive studies using larger samples and more robust sampling methods.

Despite these limitations, the results from this study gives a first glimpse into the role of explicit knowledge and learning in CBT and how treatment content is related to experiences of therapeutic change. Hopefully, further qualitative research can help practitioners how to structure ICBT for different clients and provide more insights on the active components of ICBT.

## Conclusions

This study indicates that clients can remember and apply CBT principles 6 months after treatment. Some explicitly recalled concrete CBT strategies that they still used and applied in their everyday life, others tended to remember treatment content in a more general, vague, way while still reporting the treatment had been helpful. The results underline the need to further explore explicit knowlege of ICBT, and to evaluate to what extent explicit knowledge are of importance for treatment improvement. Further studies on the role of knowledge and memory of ICBT for adolescents would be useful.

## Supplementary information

**Additional file 1.**

## Data Availability

The quotes presented were derived from transcribed interviews containing sensitive data that cannot be made publicly available.

## Abbreviations

ICBT: Internet-based cognitive behavioural therapy
CBT: Cognitive behavioural therapy
