# Supplementary material for: Knowledge gain and usage of knowledge learned during internet-based CBT treatment for adolescent depression - a qualitative study
Source: BMC Psychiatry. 2020 Sep 10;20:441. doi: 10.1186/s12888-020-02833-4 (PMC7488317; doi:10.1186/s12888-020-02833-4)
Supplement: Supplementary file 1 — Additional file 1. [file 12888_2020_2833_MOESM1_ESM.docx]

**Interview guide**

- Introduce yourself and state the purpose of the study
- Ask for informed consent
- Start interview with something like *“Now I will ask a couple of questions that may feel a little difficult to answer instantly. Give yourself some time to think. No answers are wrong or weird."*
- Ask the following questions:

***1. Do you remember what you learned from the treatment?***

***2. Have you used what you learned during the treatment and if so, in what way?***

***3. Has the knowledge helped you in your everyday life and if so, in what way?***

***4. Have you learned something that you have found unhelpful?***

For non-specific and vague answers, ask follow-up questions such as:

*- Can you tell me something more about that?*

*- Can you give a concrete example?*

*- Give yourself a moment. If you think back, you went through a treatment containing modules with texts and exercises each week. Did you learn something from these modules*?”

*- you read a lot of information about your problems and strategies, talked with your therapists and were asked to reflect and do a range of tasks, do you remember something you learned from any of these parts?*

Ask at least one more question before asking the next question; for example*, is there anything more you remember about what you learned from the treatment?*

Reflect, confirm, find ways to create a relationship if the person is perceived as shy or insecure.

Ask a question if there is anything more the person would like to add.

Thank the person for his/her participation. End interview.
